# Decision Curve Analysis of In-Hospital Mortality Prediction Models: The Relative Value of Pre- and Intraoperative Data For Decision-Making

**DOI:** 10.1213/ANE.0000000000006874

**Published:** 2024-02-05

**Authors:** Markus Huber, Corina Bello, Patrick Schober, Mark G. Filipovic, Markus M. Luedi

**Affiliations:** From the * Department of Anaesthesiology and Pain Medicine, Inselspital, Bern University Hospital, University of Bern, Bern, Switzerland; † Department of Anesthesiology, Amsterdam University Medical Centres, Vrije Universiteit Amsterdam, Amsterdam, the Netherlands.

## Abstract

**BACKGROUND:**

Clinical prediction modeling plays a pivotal part in modern clinical care, particularly in predicting the risk of in-hospital mortality. Recent modeling efforts have focused on leveraging intraoperative data sources to improve model performance. However, the individual and collective benefit of pre- and intraoperative data for clinical decision-making remains unknown. We hypothesized that pre- and intraoperative predictors contribute equally to the net benefit in a decision curve analysis (DCA) of in-hospital mortality prediction models that include pre- and intraoperative predictors.

**METHODS:**

Data from the VitalDB database featuring a subcohort of 6043 patients were used. A total of 141 predictors for in-hospital mortality were grouped into preoperative (demographics, intervention characteristics, and laboratory measurements) and intraoperative (laboratory and monitor data, drugs, and fluids) data. Prediction models using either preoperative, intraoperative, or all data were developed with multiple methods (logistic regression, neural network, random forest, gradient boosting machine, and a stacked learner). Predictive performance was evaluated by the area under the receiver-operating characteristic curve (AUROC) and under the precision-recall curve (AUPRC). Clinical utility was examined with a DCA in the predefined risk preference range (denoted by so-called treatment threshold probabilities) between 0% and 20%.

**RESULTS:**

AUROC performance of the prediction models ranged from 0.53 to 0.78. AUPRC values ranged from 0.02 to 0.25 (compared to the incidence of 0.09 in our dataset) and high AUPRC values resulted from prediction models based on preoperative laboratory values. A DCA of pre- and intraoperative prediction models highlighted that preoperative data provide the largest overall benefit for decision-making, whereas intraoperative values provide only limited benefit for decision-making compared to preoperative data. While preoperative demographics, comorbidities, and surgery-related data provide the largest benefit for low treatment thresholds up to 5% to 10%, preoperative laboratory measurements become the dominant source for decision support for higher thresholds.

**CONCLUSIONS:**

When it comes to predicting in-hospital mortality and subsequent decision-making, preoperative demographics, comorbidities, and surgery-related data provide the largest benefit for clinicians with risk-averse preferences, whereas preoperative laboratory values provide the largest benefit for decision-makers with more moderate risk preferences. Our decision-analytic investigation of different predictor categories moves beyond the question of whether certain predictors provide a benefit in traditional performance metrics (eg, AUROC). It offers a nuanced perspective on for whom these predictors might be beneficial in clinical decision-making. Follow-up studies requiring larger datasets and dedicated deep-learning models to handle continuous intraoperative data are essential to examine the robustness of our results.

**KEY POINTS**

- **Question:** What is the relative importance of pre- and intraoperative data for clinical decision-making based on in-hospital mortality prediction models?
- **Findings:** Conditional on the framework of this study, preoperative data provide the largest decision-related benefit while summarized intraoperative data provide only limited added value for clinical decision-making.
- **Meaning:** Depending on individual risk preferences when considering clinical actions based on predicted mortality risks, clinicians with very risk averse preferences should examine preoperative demographics, comorbidities and surgery-related data whereas preoperative laboratory measurements provide the largest decision support for clinicians with more moderate risk preferences. However, the benefit of intraoperative data for decision-making might be underestimated here as more advanced deep-learning modeling approaches are needed to examine the full decision-related benefit of continuous, intraoperative data.

Clinical prediction modeling plays an increasingly important role in clinical care providing both diagnostic and prognostic information to the clinician, for example, for better risk stratification and to facilitate decision-making to counter those risks.^1^ A major area of relevance for prediction modeling is the risk assessment of postoperative death: despite continuous improvements in perioperative care delivery over the past decades, postoperative mortality remains common and is estimated to lie between 0.5% - 3%, albeit with large variability between countries.^2,3^

Over the past decades, multiple prediction modeling frameworks and scores have been developed to estimate the postoperative risk of death.^4,5^ Modeling frameworks that are based on preoperative data only include, for example, the Risk Stratification Index 3.0, the Surgical Outcome Risk Tool (SORT) and the Preoperative Score to Predict Postoperative Mortality (POSPOM).^6–8^ Recent efforts have focused on assessing the relative benefits of pre- and intraoperative data for mortality prediction.^9–11^ In this context, the ability of modern deep-learning methods and natural language processing tools has been leveraged to incorporate continuous intraoperative data in the prediction models.^12–15^

The actual clinical utility of current mortality prediction models for decision-making has received less attention compared to the analysis of the models’ discriminatory abilities. Traditionally, the performance of these models––as well as the relative benefits of pre- and intraoperative data––was primarily assessed using traditional discrimination metrics such as the area under the receiver-operating characteristic curve (AUROC). However, determining the way in which prediction models can improve care delivery and disease management cannot be based on inspecting model discrimination or model calibration alone.^16,17^ In this regard, decision curve analysis (DCA) provides a way to address the clinical utility of prediction models in terms of diagnostic and prognostic strategies beyond traditional performance metrics.^18^ Previous studies used DCA with mortality prediction models using various patient characteristics, vital signs, and risk scores collected preoperatively or during hospitalization.^19–21^ However, a detailed analysis of the relative decision-related benefits of pre- and intraoperative data for in-hospital mortality prediction is still missing.

We therefore built a suite of prediction models for in-hospital mortality based on different pre- and intraoperative predictors. We hypothesized that pre- and intraoperative predictors contribute equally to the net benefit (NB) in a DCA of in-hospital mortality prediction models that include pre- and intraoperative predictors. We tested the hypothesis by comparing the decision curves of the set of prediction models and by computing ratios of NB derived with prediction models featuring either pre- or intraoperative predictors compared to the NB from a prediction model featuring both pre- and intraoperative predictors.

## METHODS

This modeling study uses a publicly available dataset that aims to support researchers in studying and developing new medical artificial intelligence algorithms called VitalDB.^22^ The need for a formal request to the local ethics committee was waived (Cantonal Ethics Commission of Bern, Bern, CH - BASEC-Nr: Req-2023-00805) as the use of the data does not fall within the federal Human Research Act.

### Data Source

The VitalDB dataset includes 6388 noncardiac (general, thoracic, urological, and gynecological) surgery patients who underwent routine or emergency surgery at the Seoul National University Hospital, Seoul, Korea, from August 2016 to June 2017.^22^ The reason for using a dataset from an external institution lies in the high quality of the VitalDB set in terms of the broad range of the collected variables and data documentation. In addition, the stated purpose of the VitalDB dataset is ideally suited for the aim of this study.

The full dataset was retrieved with the VitalDB Web API (https://vitaldb.net/dataset/?query=api) and contained scalar values and time-series data. For time-series data, the median values over the course of the operation were used to facilitate the model-building process, using the *opstart* and *opend* variables that are available for each patient; the limitations of this approach are explored in the Discussion. A flow chart describing the patient and predictor selection process is provided in Supplemental Digital Content, Supplemental Figure SM1, http://links.lww.com/AA/E689: only patients under general anesthesia and variables with <66% missing values were selected, resulting in 6043 patients and 141 predictors for the primary outcome, in-hospital mortality. The set of predictors were grouped into the following categories: preoperative demographics, comorbidities and intervention characteristics, preoperative laboratory measurements, intraoperative monitoring data and intraoperative laboratory measurements, drugs, and fluid administration.

In terms of descriptive statistics, categorical predictors are summarized using counts and frequencies. Numerical predictors are summarized with mean and standard deviation in the case of normally distributed variables and with median and interquartile range (IQR) otherwise. For exploratory purposes only, unadjusted group comparisons with respect to in-hospital mortality are shown in the summary tables; for categorical predictors, group comparisons are based on the χ^2^ test or Fisher exact test. For numerical variables, group comparisons are based on Student *t* test for normally distributed variables and on the unpaired 2-sample Wilcoxon test otherwise.

### Prediction Modelling

The study follows the network “Enhancing the QUAlity and Transparency Of Health Research” (EQUATOR)’s guidelines for transparent reporting of a multivariable prediction model for individual prognosis or diagnosis (TRIPOD).^23^ The TRIPOD checklist is provided in the Supplemental Digital Content, Supplemental Material, http://links.lww.com/AA/E689. The model-building and evaluation approach of this study is graphically illustrated in Supplemental Figure SM2, http://links.lww.com/AA/E689, and is similar to the one described in a previous study that predicted mortality in a cohort of patients with open abdomen treatment for peritonitis.^24^ The following statistical methods were used to build a clinical prediction model of in-hospital mortality: multivariable logistic regression, random forest, a single-hidden-layer neural network, and gradient boosting machine. Each of those methods was trained with predictors from each predictor category and with all available predictors. A stacked learner^25,26^ was derived by combining the cross-validated predicted probabilities of the individual base learners using a GBM. Numerical variables were standardized before model training.

In terms of model training for each method, a repeated random stratified sampling with respect to the outcome variable was performed: 60% of the available data was randomly assigned to a training set, and the remaining 40% to a test set. Five hyperparameter settings for each tunable hyperparameter were randomly sampled for each round of a 2-times repeated 2-fold cross-validation (CV). The tuneable hyperparameters for each algorithm are indicated in Supplemental Digital Content, Supplemental Figure SM2, http://links.lww.com/AA/E689. The hyperparameter combinations with the largest AUROC value were chosen as the optimal hyperparameters during the CV process. The model training and evaluation process was repeated 500 times to derive calibration and performance estimates on 500 test sets, thus providing a computationally extensive and robust internal evaluation. Given the strong imbalance in the dataset, we prioritized a high number of split-sample datasets for internal validation and aimed to avoid overfitting by choosing a relatively low number of tunable hyperparameter settings and a low number of folds in the cross-validation.

**Figure 1.**
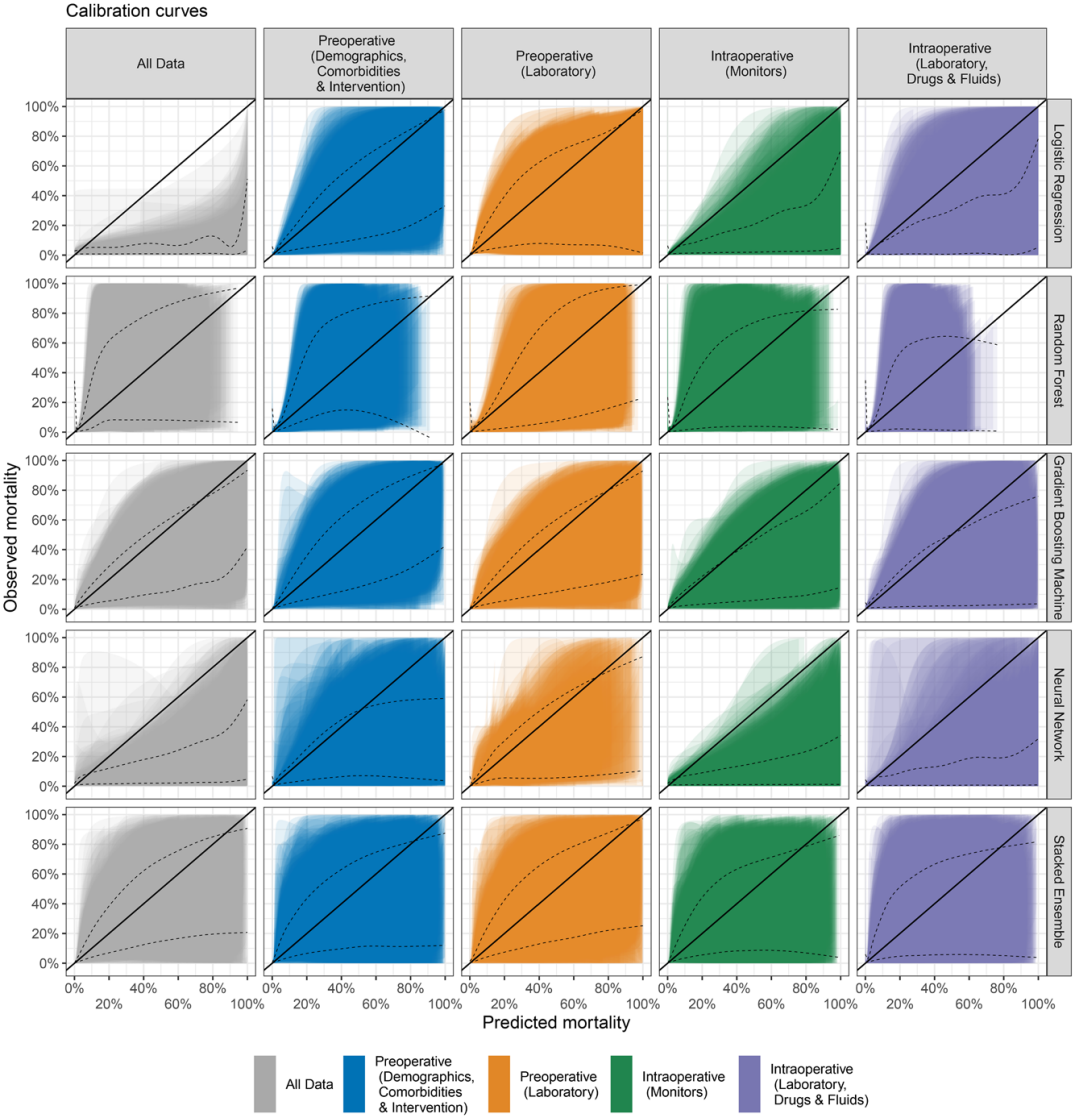
Calibration curves for the single-category prediction models and multicategory prediction models stratified according to the modeling approach. Shaded areas denote the 95% confidence intervals of the calibration belts, which estimate the relationship between true and predicted outcomes in a polynomial fashion. Black dashed lines indicate the GAM-smoothed calibration belts from the ensemble of 500 individual calibration belts. GAM indicates generalized additive model.

Calibration of the prediction models was assessed by means of calibration belts and associated 95% confidence intervals (CIs); perfect calibration is represented by the diagonal line in the calibration plot. A smoothing function (general additive model) is shown as a black dashed line in the calibration plots to denote the overall calibration across the 500-member split-sample set.

**Figure 2.**
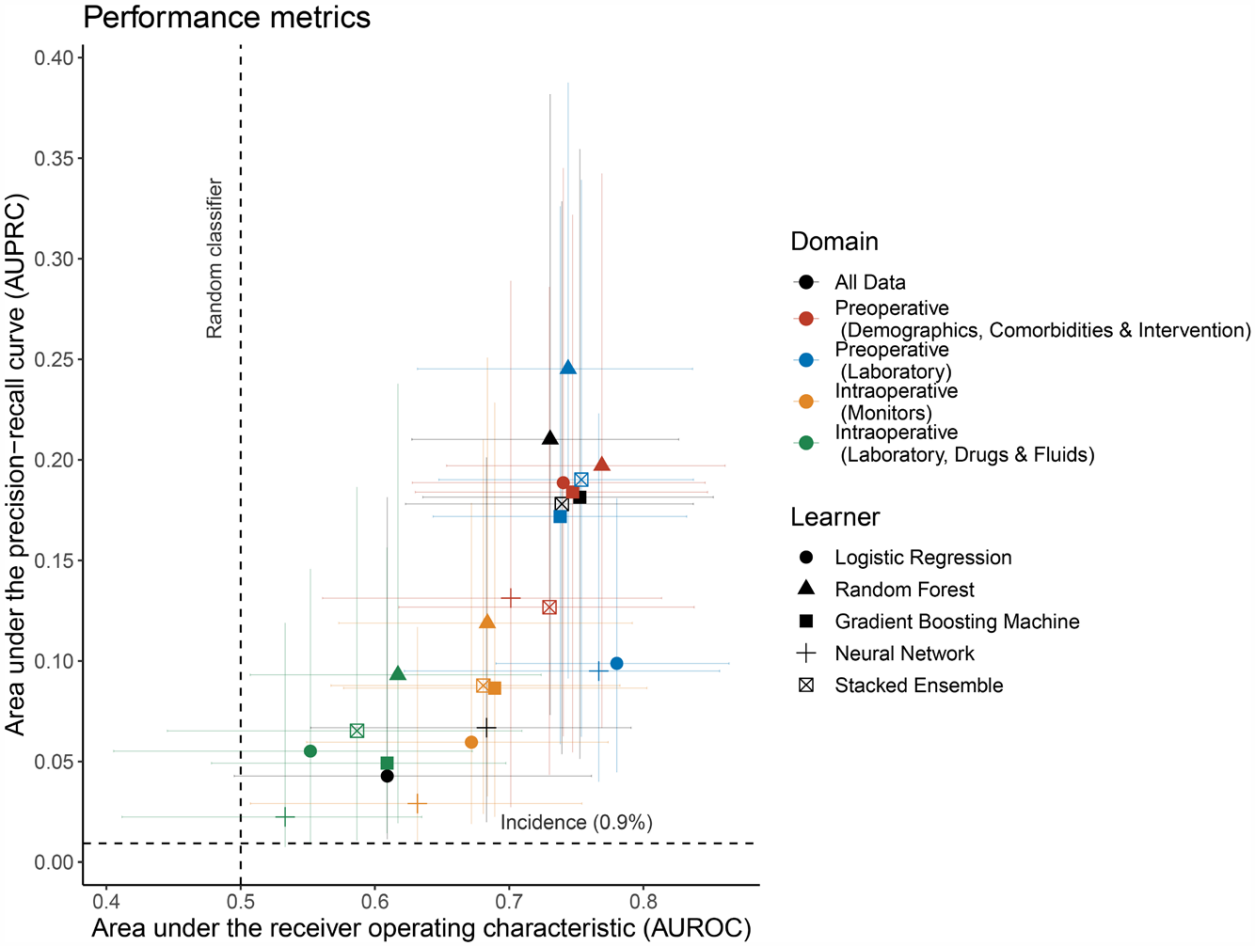
Performance metrics for single-category and multicategory prediction models. Median and 95% confidence intervals are shown based on the 500-member random split-data ensemble.

Predictive performance was evaluated by the AUROC and the area under the precision-recall curve (AUPRC). Note that in contrast to the area under the receiver-operating curve where the baseline is fixed (equal to 0.5), the baseline of the AUPRC is determined by the incidence of the positive class, which is equal to 0.9% (=56/6043) in our case and the AUPRC performance should be assessed with respect to this baseline as indicated in Figure 2. We further computed the (original and scaled) Brier Scores as performance metrics.

### Decision Curve Analysis

Given its central role in this study, we describe the DCA framework in more detail. DCA is a method to evaluate diagnostic tests and clinical prediction models for a binary outcome and centers around the concept of net benefit (NB).^18^ NB is a measure that explicitly incorporates weights for detecting true positives with the disease (TP) versus diagnosing false positives without the disease (FP). The NB is expressed in units of true positives and the NB of a true positive is compared to the harm of *w* false positives (N = sample size):^27^


$$NB=\frac{TP}{N}-w*\frac{FP}{N}$$

A further key concept in DCA is the so-called treatment threshold, which refers to the minimum probability of a disease or an event at which a clinician would take action, for example, running additional tests. Thus, the concept of the treatment threshold allows to represent different risk preferences by the clinicians (and the patients) with respect to the clinical action of interest; for example, risk-averse clinicians might choose a low threshold probability. Replacing the weight *w* by the odds at the treatment threshold (interpreted as an exchange rate between correctly versus incorrectly classified patients), we derive the following expression for the NB:^18,28^

$NB=\frac{TP}{N}-\frac{p_{T}}{1-p_{T}}*\frac{FP}{N}$, or as a function of prevalence, sensitivity, and specificity:


$$\begin{array}{l} NB=sensitivity*prevalence-\left(1-specificity\right)*\left(1-prevalence\right)*\frac{p_{T}}{1-p_{T}} \end{array}$$

In the context of this study for a prediction model with probabilistic output for a binary outcome (ie, the probability $p^{*}$ that a patients dies in our case of mortality prediction), we can calculate sensitivity and specificity at a given treatment threshold probability (denoted as $p_{T})$ by assigning a class label (ie, death) when $p^{*}\geq p_{T}$.^28^ As a concrete example for the interpretation of the treatment threshold in the setting of this study, one could think of a risk averse clinician stating, for example, “if a model based on preoperative data predicts a mortality risk of 5% or higher, I will assume that the model predicts the death of the patient and I will think of alternative treatment plans before surgery, that is, for example, not to perform the surgery or to perform additional test before surgery.” In this case the treatment threshold probability would be 5%. We can now calculate the NB of a clinical prediction model for our imbalanced dataset with a threshold probability of 5% as follows: Based on the predicted probabilities, we derive a true positive rate of 0.004 ($\frac{TP}{N}|p_{T}=0.05$) and a false positive rate of 0.002 ($\frac{FP}{N}|p_{T}=0.05$), resulting in an NB of $NB=0.004-\frac{0.05}{1-0.05}*0.002=0.0039$. In the context of a mortality prediction model, an NB of, for example, NB=0.004, could be interpreted as correctly predicting 4 patients who would die for every 1000 patients in the target population, conditional on the choice of risk preference represented by the treatment threshold $p_{T}$. The concept of NB thus provides a useful framework to compare the clinical utility of different prediction models.

Varying the treatment threshold probability over a particular range allows to draw the NB as a function of the treatment threshold probability; the resulting curves of NB are referred to as decision curves. These decision curves allow one to evaluate whether a prediction model exhibits a higher NB as the 2 default treatment strategies of “treat all” and “treat none”^28^: in our case, a “treat all” strategy simply assigns the predicted outcome death to all patients, whereas a “treat none” strategy would assume that the model predicts a survival of all patients.

We restrict the analysis of the NB of the prediction models to the threshold probability range of 0% to 20%: that is, we are much more concerned about the patients’ possible death than about unnecessary interventions for surviving patients. In addition, if the preoperative, a priori mortality risk of a patient is above a (subjective) threshold of, for example, 10% (corresponding to odds of 1:9), it is likely that additional precautions or a change of the treatment plan would have been initiated. For postoperative considerations––that is, when a prediction model with both pre- and intraoperative variables is used for decision-making––we also included higher risk preferences up to threshold probabilities of 20%. Possible preoperative interventions could constitute a change of the treatment plan (for example canceling the planned intervention), whereas possible postoperative interventions could include enhanced monitoring in the PACU or ICU or running additional postoperative laboratory tests. To aid the interpretation of the decision curves, we note that the maximum NB is equal to the in-hospital mortality incidence, which is 0.9% in our dataset.

After model training based on a particular training dataset, the mortality risk of each patient in the corresponding test dataset is predicted by the model. Decision curves are then computed with these model-derived predicted probabilities. The decision curves were computed for each base learner and the stacked learner and separately for each predictor category (eg, preoperative laboratory) within the treatment threshold range of interest (0%–20%). For each treatment threshold, the median and associated 95% CI of the median are shown since the median as a general summary measure for each threshold probability is our primary interest. For each treatment threshold, we further calculated the ratio of the NB from a prediction model with a given set of predictors (eg, preoperative laboratory values) to the NB from the prediction model featuring all available predictors to assess the relative contribution––and thus importance––of each predictor category with respect to the set of all available predictors.

### Missing Data and Statistical Software

The original dataset contained several missing data; data availability for each predictor is indicated in Tables 1 and 2 and Supplemental Digital Content, Supplemental Table SM3, http://links.lww.com/AA/E689. For each split-sample, we used a single imputation approach where missing values for binary or categorical predictors are imputed by the mode and numerical variables are imputed by the median value. The combination of a large ensemble of randomly drawn split-samples and a split-sample specific imputation approach thus allows for a robust uncertainty quantification.

**Table 1.** Patients’ and Intervention Characteristics and Preoperative Laboratory Measurements

In-hospital mortality	Survived	Died	*P* value	N
	n = 5987 (99.1%)	n = 56 (0.9%)		
Preoperative demographics, comorbidities and intervention characteristics				
Age (y)	59 [48;68]	62 [49;73]	.334	6043
Sex (female)	2993 (50.0%)	19 (33.9%)	.024	6043
Height (cm)	162 [156;169]	165 [158;170]	.456	6043
Weight (kg)	60 [53;69]	60 [51;71]	.688	6043
BMI (kg/m^2^)	23 [21;25]	23 [21;26]	.676	6043
ASA physical status			<.001	5926
I	1691 (28.8%)	9 (16.7%)		
II	3506 (59.7%)	16 (29.6%)		
III	627 (10.7%)	17 (31.5%)		
IV	36 (0.6%)	12 (22.2%)		
VI	12 (0.2%)	0 (0%)		
Emergency operation (yes)	682 (11.4%)	28 (50.0%)	<.001	6043
Surgical department			.851	6043
General surgery	4586 (76.6%)	44 (78.6%)		
Gynecology/thoracic/urology	1401 (23.4%)	12 (21.4%)		
Surgery type			.166	6043
Minor/major resection	1131 (18.9%)	6 (10.7%)		
Other	4856 (81.1%)	50 (89.3%)		
Surgical approach			.005	6043
Open	2983 (49.8%)	39 (69.6%)		
Robotic/videoscopic	3004 (50.2%)	17 (30.4%)		
Surgical position			.040	6043
Supine	3629 (60.6%)	42 (75.0%)		
Other	2358 (39.4%)	14 (25.0%)		
Preoperative hypertension (yes)	1836 (30.7%)	19 (33.9%)	.703	6043
Preoperative diabetes (yes)	632 (10.6%)	3 (5.4%)	.297	6043
Preoperative ECG			<.001	6043
Normal sinus rhythm	5922 (98.9%)	47 (83.9%)		
Other	65 (1.1%)	9 (16.1%)		
Preoperative pulmonary function			.226	6043
Normal	5011 (83.7%)	43 (76.8%)		
Obstructive/restrictive	976 (16.3%)	13 (23.2%)		
Cormack’s grade (>I)	857 (14.3%)	18 (32.1%)	<.001	6043
Airway route			<.001	6043
Oral	5902 (98.6%)	44 (78.6%)		
Other	85 (1.4%)	12 (21.4%)		
Endotracheal tube size (mm)	7.00 [7.0;7.5]	7.50 [7.0;7.5]	.200	4908
Site of IV line (1)			.842	5979
Left	4059 (68.4%)	34 (70.8%)		
Right	1872 (31.6%)	14 (29.2%)		
Site of arterial line (1)			.326	3455
Left	1149 (33.6%)	10 (25.0%)		
Right	2266 (66.4%)	30 (75.0%)		
Preoperative laboratory				
Hemoglobin (g/dL)	13.0 [11.7;14.2]	11.2 [9.1;13.0]	<.001	5724
Platelet count (x1000/mcL)	236 [192;284]	193 [105;270]	.004	5723
Prothrombin time (%)	101 [94.0;109]	86.0 [52.8;99.2]	<.001	5677
Activated partial thromboplastin time (s)	32.1 [30.1;34.4]	34.4 [31.0;53.0]	.001	5666
Sodium (mmol/L)	140 [139;142]	138 [134;140]	<.001	5448
Potassium (mmol/L)	4.2 [3.9;4.4]	3.9 [3.8;4.3]	<.001	5450
Glucose (mg/dL)	103 [94;122]	111 [94;136]	.164	5691
Albumin (g/dL)	4.2 [3.9;4.4]	3.0 [2.7;4.1]	<.001	5698
Serum GOT (IU/L)	20 [17;26]	27 [19;88]	<.001	5703
Serum GPT (IU/L)	18 [13;27]	27 [17;94]	<.001	5705
Blood urea nitrogen (mg/dL)	14 [11;17]	15 [12;20]	.063	5704
Creatinine (mg/dL)	0.78 [0.66;0.94]	0.72 [0.50;1.01]	.133	5698
Postoperative data (not included as predictors)				
Postoperative length of ICU stay (d)	0.0 [0.0;0.0]	1.0 [0.0;16.2]	<.001	6043
Surgery duration (min)	115 [65;195]	110 [70;206]	.688	6043

Unadjusted group comparisons with respect to the primary outcome in-hospital mortality are shown for exploratory purposes. Data availability is shown for each variable.

Abbreviation: ASA, American Society of Anesthesiologists; BMI, body mass index; ECG, electrocardiogram; GOT, glutamic oxaloacetic transaminase; GPT, glutamic pyruvic transaminase; ICU, intensive care unit; IV, intravenous.

**Table 2.** Summary Statistics of Intraoperative Laboratory Values, Intraoperative Drugs, and Fluid Administration

In-hospital mortality	Survived	Died	*p*	N
	n = 5987 (99.1%)	n = 56 (0.9%)		
Laboratory				
Glucose (mg/dL)	115 [100;138]	118 [110;131]	.365	2953
Bicarbonate (mmol/L)	26 [24;28]	23 [21;27]	.001	2960
Hematocrit (%)	34 [30;38]	30 [27;33]	.002	2959
Ionized Ca^2+^ (mmol/L)	1.13 [1.09;1.16]	1.15 [1.12;1.20]	.027	2960
Potassium (mmol/L)	3.6 [3.4;3.9]	3.7 [3.5;4.0]	.355	2963
Lactate (mmol/L)	1.0 [0.8;1.4]	1.3 [0.9;3.6]	.003	2941
Sodium (mmol/L)	134 [133;136]	135 [132;137]	.619	2963
Partial pressure of CO_2_ (mm Hg)	42 [39;45]	41 [38;44]	.303	2960
pH (–)	7.40 [7.36;7.43]	7.37 [7.33;7.42]	.063	2961
Partial pressure of O_2_ (mm Hg)	161 [126;201]	168 [126;203]	.701	2961
Arterial oxygen saturation (%)	99.0 [99.0;100]	99.5 [99.0;100]	.818	2960
Drugs				
Propofol bolus (mg)	0 [0;100]	0 [0;60]	.114	6043
Midazolam (mg)	0 [0;0]	0 [0;0]	<.001	6043
Fentanyl (µg)	0 [0;0]	0 [0;0]	.763	6043
Rocuronium (mg)	70.0 [50.0;100]	70.0 [50.0;92.5]	.338	6043
Vecuronium (mg)	0 [0;0]	0 [0;0]	.737	6043
Ephedrine (mg)	5 [0;10]	0 [0;5]	.028	6043
Phenylephrine (µg)	0 [0;0]	0 [0;7.5]	.007	6043
Epinephrine (µg)	0 [0;0]	0 [0;0]	<.001	6043
Calcium chloride (mg)	0 [0;0]	0 [0;300]	<.001	6043
Fluids				
Estimated blood loss (mL)	150 [50.0;300]	250 [100;1000]	.003	3897
Urine output (mL)	160 [90.0;300]	200 [53.8;311]	.937	3693
RBC transfusion (units)	0 [0;0]	0 [0;0]	<.001	6043
FFP transfusion (units)	0 [0;0]	0 [0;0]	<.001	6043
Crystalloids (mL)	700 [400;1400]	1025 [400;1875]	.177	5713
Colloids (mL)	0 [0;0]	0 [0;0]	.015	6043

Unadjusted group comparisons with respect to the primary outcome in-hospital mortality are shown for exploratory purposes. Data availability is shown for each variable. Note that there are several zero-inflated variables, for example, epinephrine administration (µg), which were not dichotomized as the original scale of the variables were used in model training.

Abbreviation: RBC, red blood cells.

In terms of statistical software, the prediction models were computed with the *caret* package^29^ and decision curves were calculated with the *dcurves* package.^30^ Bootstrapped 95% CIs for the median (999 replicates) are computed with the *DescTools*^31^ package. All computations were performed with R version 4.0.5.^32^

## RESULTS

### Cohort Description

The demographic and procedural characteristics as well as the preoperative laboratory measurements of the cohort considered here are shown in Table 1. Median age was 59 years (IQR, 48–68 years) and 49.8% were female. Patients featured a median BMI of 23.1 kg/m^2^ (IQR, 20.9–25.3 kg/m^2^) and the majority were classified with American Society of Anesthesiologists (ASA) physical status II (59.4%). Overall, there were 710 emergency procedures (11.7%). Surviving patients featured, for example, higher preoperative hemoglobin values (13.0 g/dL, IQR, 11.7–14.2 g/dL, vs 11.2 g/dL, IQR, 9.1–13.0 g/dL, unadjusted *P* < .001). Summary measures for intraoperative laboratory measurements, drug, and fluid administration are shown in Table 2. Surviving patients showed, for example, lower intraoperative lactate values (1.00 mmol/L, IQR, 0.25–1.40 mmol/L, vs 1.32 mmol/L, IQR, 0.90–3.55 mmol/L, unadjusted *P* = .003). Supplemental Digital Content, Supplemental Table SM3, http://links.lww.com/AA/E689 shows the summary measures of the 83 monitor-derived predictors, indicating, for example, a median bispectral index value (BIS) of 40.7 (IQR, 36.7–44.7).

### Calibration

Figure 1 shows the calibration belts for each modeling approach and predictor category, relating the model-predicted mortality to the observed mortality in the cohort. Perfect calibration is denoted by the diagonal 1:1 line. Overall, the majority of modeling approaches tend to overestimate the mortality risk, in particular for high predicted risks. In terms of modeling approaches, logistic regression and the neural network feature the largest biases. Prediction models based on a random forest generally demonstrate the smallest biases.

### Performance Metrics

The joint medians and 95% CIs of the 2 performance metrics AUROC and AUPRC for the different predictor categories and modeling approaches are shown in Figure 2; the numerical values are listed in the Supplemental Digital Content, Supplemental Table SM4, http://links.lww.com/AA/E689. The AUROC performances vary strongly both across predictor categories and across model types with a range of median AUROC values from 0.53 to 0.78. Figure 2 highlights that preoperative predictors (color-coded in red and blue) are the dominant contributors to high AUROC and AUPRC values. In terms of all predictors, the stacked ensemble features an AUROC of 0.74 (95% CI, 0.62–0.84) and an AUPRC of 0.18 (95% CI, 0.05–0.33). Overall, the random forest prediction models generally feature the strongest performance, both in terms of AUROC and AUPRC, and the highest single-category AUPRC was achieved by the random forest model when preoperative laboratory values were used as predictors (median AUPRC of 0.25, 95% CI, 0.09–0.39).

While there is a strong correlation between the 2 performance metrics in Figure 2, there are notable exceptions. Figure 2 highlights that there are prediction models with equal performance in terms of the AUROC metric but with highly different AUPRC performances, thus highlighting that for strongly imbalanced dataset such as featured in this study, considering the AUROC and AUPRC metric jointly may provide additional insights when comparing the discriminatory performance across a set of models.

### Decision Curve Analysis

**Figure 3.**
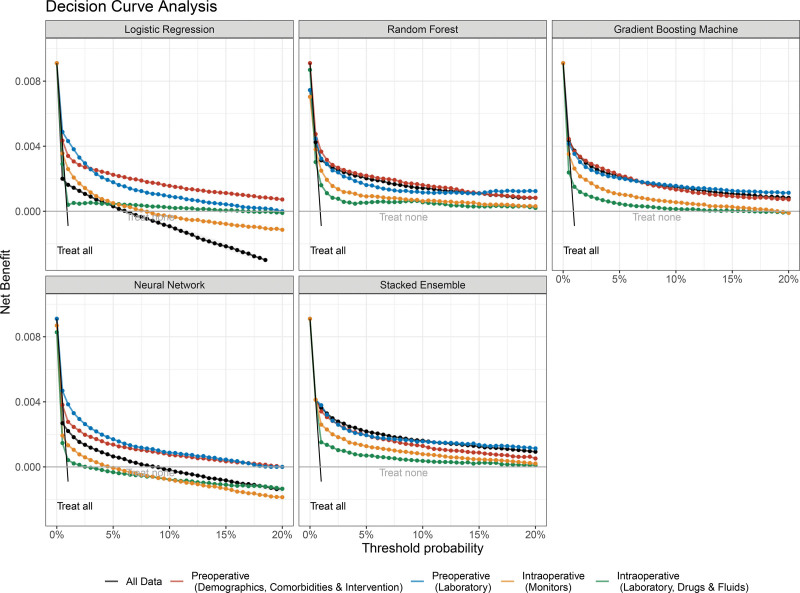
Decision curve analysis of clinical prediction models using different sets of predictor variables (color-coded). The decision curves of each modeling approach and predictor set are summarized by the median and the 95% confidence interval of the median and are compared to the 2 default strategies “treat all” and “treat none.”

Figure 3 shows the decision curves for the different modeling approaches and separately for the pre- and intraoperative predictor categories. Overall, the decision-related benefit of the prediction models for in-hospital mortality are largest for risk averse preferences within a threshold probability range between approximately 1% to 5%; the benefit generally decreases for more moderate risk preferences (eg, up to threshold probabilities of 20%). Although there are significant differences of the relative importance of different predictor categories with respect to the modeling approach––in particular regarding the set of miscalibrated models, that is, the neural networks––Figure 3 highlights that preoperative predictors provide on average larger decision-analytic benefit than summary measures of intraoperative data.

**Figure 4.**
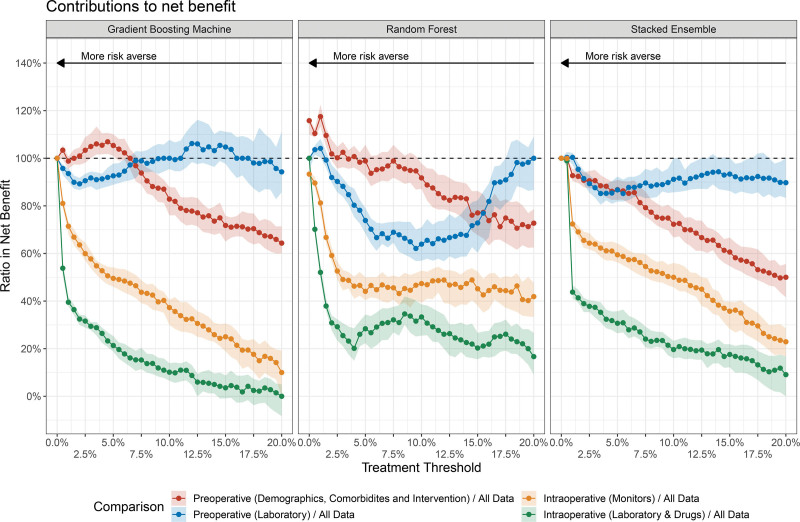
Comparison of the decision-analytic contributions of different predictor sets to overall net benefit. For each predictor set (eg, preoperative laboratory measurements) and each treatment threshold (eg, 5%), the ratio of the net benefit of a clinical prediction model using the predictor set of interest and the net benefit of a clinical prediction model using all available predictors are shown. The median and 95% confidence interval of the median of the corresponding ratios are shown.

Figure 4 provides a more detailed analysis on the relative decision-related importance of the different pre- and intraoperative predictors with respect to a prediction model featuring all available predictors. The miscalibrated prediction models based on logistic regression and the neural network are omitted for this part of the analysis. The ratios in NB highlight that for risk averse preferences (treatment thresholds up to around 5%), considering only preoperative demographics, comorbidities and surgery characteristics may provide the highest benefit––even higher than the benefit from a prediction model featuring all predictors. There is evidence that the relative importance of this routinely collected set of preoperative data decreases for more moderate risk preference and that preoperative laboratory values become increasingly important for higher, more moderate treatment thresholds. Figure 4 further highlights that summarized intraoperative predictors––both from laboratory measurements and from monitors––provide only minor benefit relative to preoperative predictors.

## DISCUSSION

The primary objective of this decision-analytic modeling study was not to derive a particularly high-performant clinical prediction model of in-hospital mortality; several high-performant models of postoperative mortality have been built over the past years: the performance of these models are higher with respect to the AUROC and the models are based on much larger cohorts.^4^ Rather, the objective was to examine our hypothesis that pre- and intraoperative data contribute equally to the NB in a DCA for in-hospital mortality prediction models that feature both pre- and intraoperative predictors. We note that previous studies already focused on assessing the relative benefits of pre- and intraoperative data for mortality prediction,^9–11^ however, to our knowledge, this is the first study that moves this comparison beyond traditional performance metrics such as the AUROC.

Within the framework of this study––for example, the choice of dataset, variable and patient selection as well as the modeling approaches––the results highlight that preoperative demographics, comorbidities and surgery-related variables generally provide the highest decision-related benefit for clinical decision-makers with risk averse preferences (treatment thresholds below ca. 5%). We find some evidence that preoperative laboratory become increasingly important for decision-making with respect to in-hospital mortality for higher treatment thresholds (eg, less risk averse conditions). In this context, a machine learning model (random forest) highlights the importance of preoperative laboratory measurements: this model features highest AUPRC metric and the AUPRC is suggested to be more informative than the AUROC for highly imbalanced data such as present in this study.^33^ Overall, the study highlights that with respect to in-hospital mortality, the more risk averse a decision-maker is, the larger is the benefit from considering a prediction model. We also found that summarized intraoperative data provide only limited decision-related benefit.

In contrast to prediction models that use more advanced machine learning methods to handle continuous intraoperative data,^13,14^ the prediction models developed here cannot be used for intraoperative decision-making because we used summarized forms of the continuous intraoperative data. This constitutes a major limitations for the generalizability of our results. More advanced deep-learning methods (eg, a convolutional neural network) were beyond the scope of this study and it is likely that our approach underestimates the decision-related benefit of intraoperative monitoring and laboratory data. Follow-up studies could thus compute time-dependent decision curves––that is, using summary measures after 15min, 30min etc.––to overcome the information loss associated with summarized intraoperative data. While this approach would be feasible for intraoperative monitoring data, it would pose challenges with respect to intraoperative laboratory data as a sufficiently high and consistent sampling rate of laboratory measurements would be required. In this context, a recent study of mortality prediction after cardiac surgery suggests that the value of continuous, high-dimensional intraoperative might be limited as postoperative markers might be the dominant factors for mortality risk prediction, even if continuous intraoperative data is modeled with a recurrent neural network able to handle time-series data.^14^

Overall, our decision-analytic investigation of different predictor categories allows moving beyond the narrow question of *if* certain predictors provide a performance benefit in terms of traditional performance measures (eg, AUROC) towards a more nuanced perspective of *for whom* they might provide a benefit for clinical decision-making. By providing evidence on which clinical factors are most relevant for the clinical implications of the prediction models, the approach outlined in this study could constitute a step towards more clinically interpretable prediction models.

Further, the observed lack of implementation of risk prediction models in the daily clinical routine practice^4,6^ could also be partly overcome by using more decision-analytic approaches in clinical routine. Consider the following case: a clinician examines the postoperative in-hospital mortality risk for a given patient after surgery using a prediction model that is based on pre- and intraoperative data. Based on multiple factors, that is, the patient’s history, the intervention and the level of clinical experience, the clinician adopts a moderate risk approach with a treatment threshold of 8%. Now assume that the prediction model, for example, a stacked learner (Figure 4), estimates the postoperative mortality risk of this patient to be 10%: since the predicted risk is above the treatment threshold, the clinician would contemplate on different actions. Now, if the clinician knows that preoperative laboratory measurements contribute the largest decision-analytic benefit for her choice of treatment threshold, she could, for example, reinvestigate the preoperative laboratory results and run additional postoperative laboratory tests based on this examination, for example for a biomarker that is costly or difficult to obtain. This example demonstrates the clinical utility of decision-analytic approaches such as outlined in this study by providing a clinical perspective of prediction models beyond predicted probabilities and performance metrics.

This study features some inherent limitations. First, we emphasize that the results are subject to the underlying dataset, the modeling approach and the choice of threshold ranges. Second, this study uses data from a single center, and even from an external institution, and an external validation of the results is required to provide a more robust quantification of our findings. Thus, generalizability remains a key challenge of this study and of mortality risk prediction models in general.^34^ Third, the strong miscalibration found in some prediction models might limit their clinical utlity.^35^ Follow-up studies could include dedicated methods such as Platt scaling and isotonic regression to improve calibration. Fourth, we did not use methods for feature explanation in machine learning models which have been previously used for mortality prediction models.^36^ Since we focused on clinical interpretability rather than statistical feature explanation, such an analysis could be tackled in a follow-up study.

Fifth, in-hospital mortality is a rare event and the topic of class imbalance was primarily addressed by supplementing the AUROC metric with the AUPRC measure. No data sampling methods to synthetically augment the raw data such as SMOTE^37^ were used. However, it was recently shown that these methods might result in miscalibrated (logistic) models and may even worsen performance.^38^ Sixth, given the high-dimensional dataset to predict mortality, some of the associations of the trained machine learning algorithms might be spurious, and causal inference methods are required to understand the causal effects which may help in better contemplating on the clinical actions that could be taken based on the predicted risks.^39,40^

Additionally, the dataset features a large degree of missing data, in particular with respect to laboratory measurements during surgery. Reducing the amount of allowed missingness in the prediction selection, that is, only 5% instead of 66% missing data for a variable, would likely remove all information related to intraoperative laboratory measurements, which might result in biased estimates. Therefore, we allowed a large degree of missingness in the predictors and accounted for this by using a large ensemble of random split-data sets and by performing imputation independently for each training and test set.

## CONCLUSIONS

In summary, when it comes to predicting in-hospital mortality and subsequent decision-making, preoperative demographics, comorbidities, and surgery-related data provide the largest benefit for clinicians with risk-averse preferences, whereas preoperative laboratory values provide the largest benefit for decision-makers with more moderate risk preferences. Our decision-analytic investigation of different predictor categories allows moving beyond the narrow question of *if* certain predictors provide a performance benefit in terms of traditional performance measures towards a more nuanced perspective of *for whom* they might provide a benefit for clinical decision-making. Follow-up studies requiring both large datasets with less missing data and dedicated deep-learning models to handle continuous intraoperative data are essential to examine the robustness of our results. In addition, these studies could also include postoperative data in this decision-analytic framework.

## DISCLOSURES

**Name:** Markus Huber, Dr. sc. ETH.

**Contribution:** This author helped in conception or design of the work, the acquisition, analysis, and interpretation of data as well as drafting and revising the article.

**Name:** Corina Bello, MD.

**Contribution:** This author helped in the clinical interpretation of data as well as drafting and revising the article.

**Name:** Patrick Schober, MD, MMedStat.

**Contribution:** This author helped in the clinical and statistical interpretation of data as well as revising the article.

**Name:** Mark G. Filipovic, MD.

**Contribution:** This author helped in the clinical interpretation of data as well as drafting and revising the article.

**Name:** Markus M. Luedi, MD, MBA.

**Contribution:** This author helped in conception or design of the work and the interpretation of data as well as drafting and revising the article.

**This manuscript was handled by:** Thomas R. Vetter, MD, MPH, MFA.

## Supplementary Material
