# Supplementary material for: Decision Curve Analysis of In-Hospital Mortality Prediction Models: The Relative Value of Pre- and Intraoperative Data For Decision-Making
Source: Anesth Analg. 2024 Feb 5;139(3):617–28. doi: 10.1213/ANE.0000000000006874 (PMC12757125; doi:10.1213/ANE.0000000000006874)
Supplement: Supplementary file 1 [file ane-139-617-s001.docx]

**SUPPLEMENTARY MATERIAL**

**Decision curve analysis of in-hospital mortality prediction models: the relative value of pre- and intraoperative data for decision-making**

Markus HUBER, Dr. sc. ETH^1*^; Corina BELLO, MD^1^, Patrick SCHOBER, MD, MMedStat^2^, Mark G. FILIPOVIC, MD^1^, Markus M. LUEDI, MD, MBA^1^

^1^ Department of Anaesthesiology and Pain Medicine, Inselspital, Bern University Hospital, University of Bern, Freiburgstrasse, 3010, Bern, Switzerland.

^2^ Department of Anesthesiology, Amsterdam University Medical Centres, Vrije Universiteit Amsterdam, Amsterdam, the Netherlands.

*Corresponding author:

Markus Huber, Dr. sc. ETH, Department of Anesthesiology and Pain Medicine, Inselspital, Bern University Hospital, University of Bern, Bern, Switzerland, Freiburgstrasse 10, 3010 Bern, Switzerland, Tel: +41 31 664 12 15, Email: markus.huber@insel.ch

**
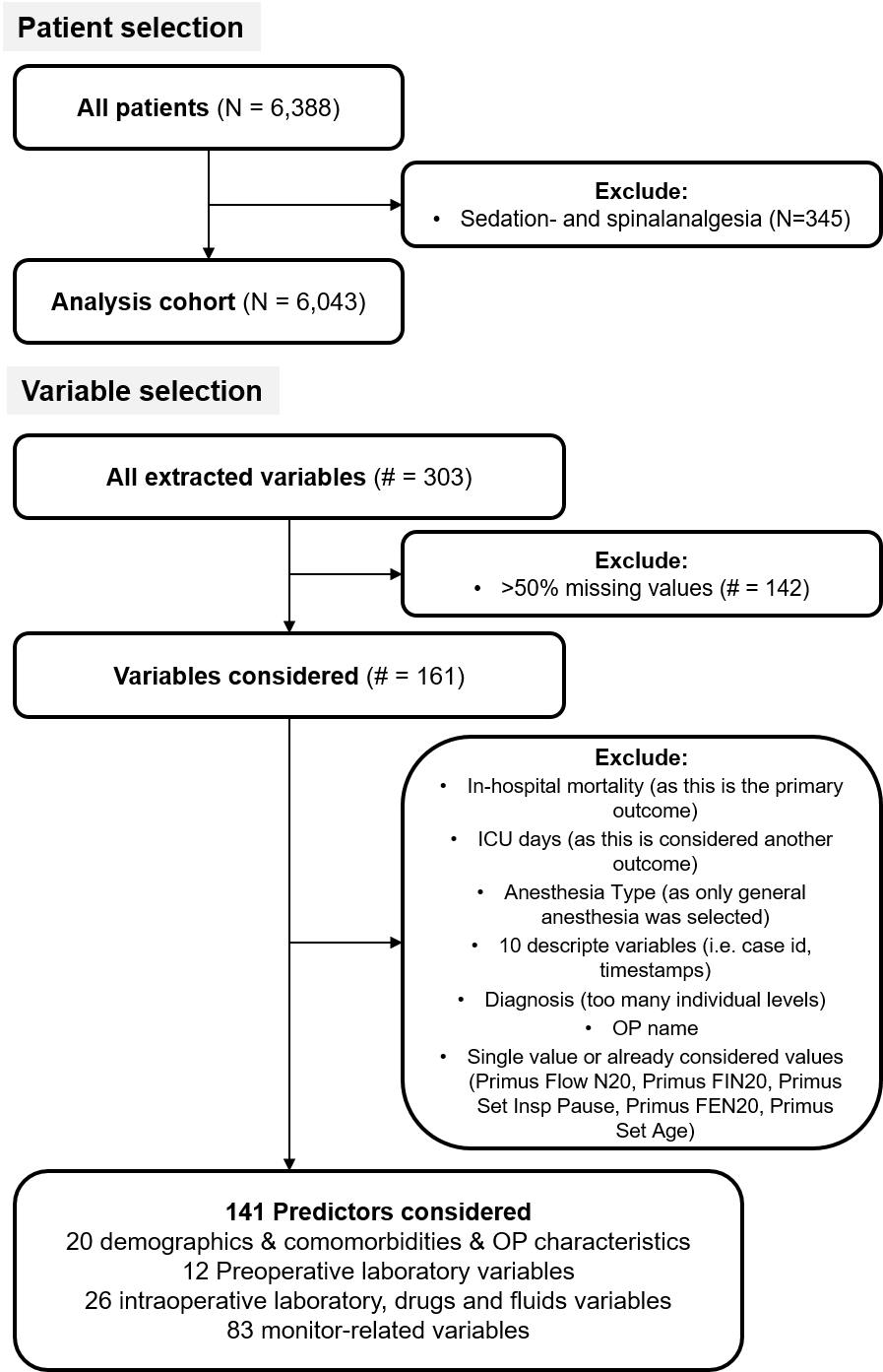
**

**Supplementary Figure SM1.** Flow chart of patient and predictor selection.

**
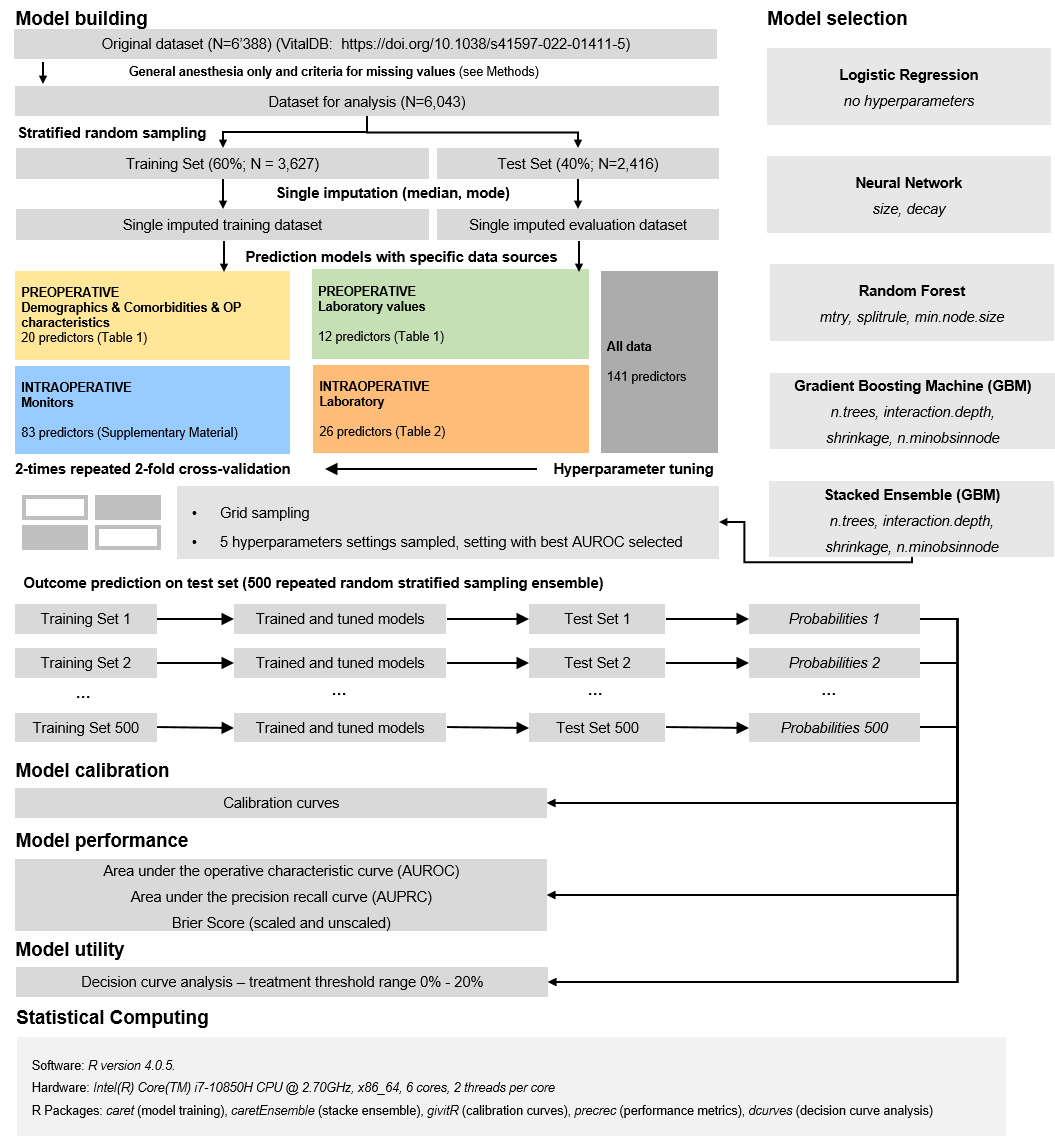
**

**Supplementary Figure SM2.** Model building and evaluation approach in this study. A detailed description of the approach is provided in the Methods section.

| **In-hospital mortality** | | **All patients** | | **Survived** | | **Died** | | ***p*** | | **N** | |
| --- | --- | --- | --- | --- | --- | --- | --- | --- | --- | --- | --- |
|  |  | ***N=6,043 (100%)*** | | ***N=5,987 (99.1%)*** | | ***N=56 (0.9%)*** | |  | |  | |
| *Primus Anesthesia Machine* | |  | |  | |  | |  | |  | |
| Airway compliance (mL/mbar) | | 30.8 [24.2;38.1] | | 30.8 [24.2;38.1] | | 30.8 [20.2;37.7] | | 0.192 | | 5,955 | |
| Inspiratory sevoflurane pressure (kPa) | | 1.60 [0.00;2.20] | | 1.60 [0.00;2.20] | | 1.15 [0.00;1.78] | | 0.602 | | 3,417 | |
| Set fraction of inspired O2 (%) | | 40.0 [40.0;50.0] | | 40.0 [40.0;50.0] | | 50.0 [40.0;67.5] | | <0.001 | | 5,967 | |
| Capnography wave (mmHg) | | 36.2 [31.7;38.8] | | 36.2 [31.7;38.8] | | 32.6 [25.4;37.5] | | <0.001 | | 5,987 | |
| Flow rate of O2 (mL/min) | | 481 [481;734] | | 481 [481;734] | | 734 [481;1493] | | 0.001 | | 5,298 | |
| Respiratory rate based on capnography (/min) | | 14.0 [13.0;16.0] | | 14.0 [13.0;16.0] | | 15.0 [13.0;18.0] | | 0.116 | | 5,976 | |
| Tidal volume (mL) | | 386 [335;443] | | 386 [335;444] | | 376 [311;439] | | 0.265 | | 5,964 | |
| End-tidal CO2 (mmHg) | | 35.0 [33.0;37.0] | | 35.0 [33.0;37.0] | | 33.0 [31.0;35.0] | | <0.001 | | 5,973 | |
| Minute volume (L) | | 5.50 [4.70;6.40] | | 5.50 [4.70;6.40] | | 5.40 [4.43;6.42] | | 0.328 | | 5,967 | |
| Set inspiratory time (sec) | | 1.40 [1.30;1.60] | | 1.40 [1.30;1.60] | | 1.40 [1.10;1.60] | | 0.131 | | 5,930 | |
| Mean airway pressure (mbar) | | 7.00 [4.00;9.00] | | 7.00 [4.00;9.00] | | 8.00 [4.50;9.50] | | 0.194 | | 5,977 | |
| Flow rate of air (mL/min) | | 1518 [1265;1519] | | 1518 [1266;1519] | | 1266 [507;1519] | | 0.001 | | 5,295 | |
| Peak inspiratory pressure (mbar) | | 17.0 [14.0;21.0] | | 17.0 [14.0;21.0] | | 18.0 [15.0;23.0] | | 0.217 | | 5,947 | |
| Set peak inspiratory pressure (mbar) | | 40.0 [35.0;40.0] | | 40.0 [35.0;40.0] | | 40.0 [35.0;40.0] | | 0.225 | | 5,900 | |
| Minimum alveolar concentration of volatile (-) | | 0.00 [0.00;0.90] | | 0.00 [0.00;0.90] | | 0.40 [0.00;0.85] | | 0.366 | | 5,981 | |
| Set fresh gas flow (mL/min) | | 2000 [2000;2000] | | 2000 [2000;2000] | | 2000 [2000;2000] | | <0.001 | | 5,967 | |
| Set respiratory rate (mbar) | | 14.0 [13.0;16.0] | | 14.0 [13.0;16.0] | | 15.0 [13.0;18.0] | | 0.145 | | 5,921 | |
| Airway pressure wave (hPa) | | 5.24 [0.90;5.90] | | 5.24 [0.90;5.90] | | 5.45 [0.90;6.32] | | 0.251 | | 5,974 | |
| Plateau pressure (mbar) | | 16.0 [13.0;19.0] | | 16.0 [13.0;19.0] | | 17.0 [14.0;20.8] | | 0.094 | | 5,936 | |
| Set tidal volume in liter (L) | | 0.40 [0.35;0.45] | | 0.40 [0.35;0.45] | | 0.40 [0.35;0.43] | | 0.971 | | 5,901 | |
| *Solar8000 Patient Monitor* | |  | |  | |  | |  | |  | |
| Fraction of inspired O2 (%) | | 36.0 [35.0;45.0] | | 36.0 [35.0;45.0] | | 44.0 [36.0;73.0] | | <0.001 | | 5,976 | |
| Positive end expiratory pressure (PEEP; mbar) | | 4.00 [0.00;5.00] | | 4.00 [0.00;5.00] | | 5.00 [0.00;5.00] | | 0.082 | | 5,937 | |
| Fraction of expired O2 (%) | | 31.0 [29.0;40.0] | | 31.0 [29.0;40.0] | | 39.0 [30.0;68.0] | | <0.001 | | 5,973 | |
| Ambient pressure (mbar) | | 1007 [1000;1014] | | 1007 [1000;1014] | | 1006 [999;1012] | | 0.186 | | 5,980 | |
| Set positive end expiratory pressure (PEEP; mbar) | | 5 [0;5] | | 5 [0;5.] | | 5 [0;5] | | 0.082 | | 5,924 | |
| Ventilator leakage (mL/min) | | 16.0 [11.0;23.0] | | 16.0 [11.0;23.0] | | 15.5 [9.75;24.0] | | 0.735 | | 5,963 | |
| Inspiratory CO2 (mmHg) | | 1.00 [1.00;1.00] | | 1.00 [1.00;1.00] | | 1.00 [1.00;1.00] | | 0.550 | | 5,965 | |
| Expiratory sevoflurane pressure (kPa) | | 1.40 [0.00;1.90] | | 1.40 [0.00;1.90] | | 1.05 [0.00;1.65] | | 0.662 | | 3,422 | |
| Fraction of inspired O2 (%) | | 36.0 [35.0;45.0] | | 36.0 [35.0;45.0] | | 44.0 [36.0;69.2] | | 0.001 | | 5,954 | |
| Respiratory rate based on capnography (/min) | | 14.0 [13.0;16.0] | | 14.0 [13.0;16.0] | | 15.0 [13.0;18.0] | | 0.153 | | 5,959 | |
| Peak inspiratory pressure (from ventilator; mbar) | | 17.0 [14.0;21.0] | | 17.0 [14.0;21.0] | | 18.0 [15.0;23.2] | | 0.292 | | 5,894 | |
| Inspiratory volatile concentration (%) | | 2.30 [2.00;3.90] | | 2.30 [2.00;3.90] | | 1.75 [1.40;2.60] | | 0.006 | | 2,907 | |
| Mean airway pressure (from ventilator; mbar) | | 7.00 [4.00;9.00] | | 7.00 [4.00;9.00] | | 8.00 [4.00;9.00] | | 0.256 | | 5,927 | |
| Non-invasive diastolic arterial pressure (mmHg) | | 66.0 [59.0;74.0] | | 66.0 [59.0;74.0] | | 66.5 [59.0;71.0] | | 0.695 | | 4,711 | |
| ST segment in lead II (mm) | | 0.10 [0.00;0.30] | | 0.10 [0.00;0.30] | | 0.10 [0.00;0.30] | | 0.921 | | 5,585 | |
| Set tidal volume in volume control mode (from ventilator; mL) | | 400 [350;450] | | 400 [350;450] | | 400 [355;435] | | 0.433 | | 4,162 | |
| Heart rate (/min) | | 69.0 [62.0;79.0] | | 69.0 [62.0;79.0] | | 83.0 [66.0;105] | | <0.001 | | 6,004 | |
| Body temperature (℃) | | 36.0 [35.6;36.3] | | 36.0 [35.6;36.3] | | 35.9 [35.5;36.6] | | 0.997 | | 5,859 | |
| ST segment in lead aVR (mm) | | -0.10 [-0.10;0.00] | | -0.10 [-0.10;0.00] | | 0.00 [-0.10;0.00] | | 0.376 | | 2,786 | |
| Measured tidal volume (from ventilator; mL) | | 386 [335;444] | | 386 [335;444] | | 386 [328;436] | | 0.530 | | 5,903 | |
| Inspiratory time (from ventilator; sec) | | 1.40 [1.30;1.60] | | 1.40 [1.30;1.60] | | 1.40 [1.17;1.60] | | 0.279 | | 5,866 | |
| Heart rate based on plethysmography (/min) | | 70.0 [62.0;79.0] | | 70.0 [62.0;79.0] | | 85.0 [66.8;105] | | <0.001 | | 5,999 | |
| Set peak inspiratory pressure in pressure control mode (from ventilator; cmH2O) | | 40.0 [35.0;40.0] | | 40.0 [35.0;40.0] | | 40.0 [40.0;40.0] | | 0.349 | | 2,694 | |
| ST segment in lead aVL (mm) | | -0.10 [-0.10;0.00] | | -0.10 [-0.10;0.00] | | 0.00 [-0.10;0.00] | | 0.376 | | 2,773 | |
| Respiratory rate (from ventilator; /min) | | 14.0 [13.0;16.0] | | 14.0 [13.0;16.0] | | 15.0 [13.0;16.5] | | 0.337 | | 5,904 | |
| End-tidal CO2 (mmHg) | | 35.0 [33.0;37.0] | | 35.0 [33.0;37.0] | | 34.0 [31.2;35.0] | | <0.001 | | 5,971 | |
| Non-invasive systolic arterial pressure (mmHg) | | 111 [102;122] | | 111 [102;122] | | 109 [95.5;119] | | 0.455 | | 4,704 | |
| ST segment in lead I (mm) | | 0.0 [0.0;0.1] | | 0.0 [0.0;0.1] | | 0.0 [0.0;0.1] | | 0.632 | | 2,790 | |
| Diastolic arterial pressure (mmHg) | | 63.0 [57.0;68.0] | | 63.0 [57.0;68.0] | | 56.0 [49.0;64.8] | | <0.001 | | 3,488 | |
| Percutaneous oxygen saturation (%) | | 100 [99.0;100] | | 100 [99.0;100] | | 100 [99.0;100] | | 0.663 | | 6,009 | |
| Mean arterial pressure (mmHg) | | 82.0 [76.0;89.0] | | 83.0 [76.0;89.0] | | 73.0 [65.0;84.5] | | <0.001 | | 3,487 | |
| Non-invasive mean arterial pressure (mmHg) | | 82.0 [74.0;90.0] | | 82.0 [74.0;90.0] | | 81.5 [74.0;87.5] | | 0.413 | | 4,716 | |
| ST segment in lead III (mm) | | 0.00 [0.00;0.20] | | 0.00 [0.00;0.20] | | 0.00 [0.00;0.20] | | 0.823 | | 2,787 | |
| Inspiratory CO2 (mmHg) | | 1.00 [1.00;1.00] | | 1.00 [1.00;1.00] | | 1.00 [1.00;1.00] | | 0.131 | | 5,969 | |
| Minute ventilation (from ventilator; L/min) | | 5.50 [4.70;6.40] | | 5.50 [4.70;6.40] | | 5.50 [4.50;6.53] | | 0.592 | | 5,922 | |
| Systolic arterial pressure (mmHg) | | 116 [108;125] | | 116 [109;125] | | 106 [94.5;114] | | <0.001 | | 3,486 | |
| Fraction of expired O2 (%) | | 31.0 [29.0;40.0] | | 31.0 [29.0;40.0] | | 39.0 [30.0;65.2] | | 0.001 | | 5,972 | |
| ST segment in lead aVF (mm) | | 0.10 [0.00;0.20] | | 0.10 [0.00;0.20] | | 0.00 [-0.10;0.20] | | 0.366 | | 2,781 | |
| Plateau pressure (from ventilator; mbar) | | 16.0 [13.0;19.0] | | 16.0 [13.0;19.0] | | 17.0 [14.0;20.5] | | 0.178 | | 5,897 | |
| Expiratory volatile concentration (%) | | 2.00 [1.70;3.05] | | 2.00 [1.70;3.08] | | 1.60 [1.10;2.22] | | 0.009 | | 2,919 | |
| Set fraction of inspired O2 (from ventilator; %) | | 40.0 [40.0;40.0] | | 40.0 [40.0;40.0] | | 40.0 [40.0;50.0] | | 0.035 | | 2,166 | |
| *Tram-Rac 4A (SNUADC) Patient Monitor* |  | |  | |  | |  | |  | |  |
| Arterial pressure wave (mmHg) | | 77.1 [70.2;85.0] | | 77.1 [70.2;85.0] | | 70.2 [60.3;81.3] | | 0.001 | | 3,590 | |
| Plethysmography wave (-) | | 36.0 [34.4;37.9] | | 36.0 [34.4;37.9] | | 37.0 [35.6;39.1] | | 0.011 | | 5,776 | |
| ECG lead II wave (mV) | | 0.01 [-0.03;0.04] | | 0.01 [-0.03;0.04] | | 0.02 [-0.02;0.04] | | 0.632 | | 5,966 | |
| ECG lead V5 wave (mV) | | 0.02 [-0.02;0.05] | | 0.02 [-0.02;0.05] | | 0.02 [-0.02;0.04] | | 0.361 | | 3,162 | |
| *Orchestra Target-controlled infusion pump* |  | |  | |  | |  | |  | |  |
| Infusion rate (propofol 20 mg/mL; mL/hr) | | 19.0 [15.8;22.6] | | 19.0 [15.9;22.6] | | 15.2 [11.4;16.5] | | <0.001 | | 3,411 | |
| Effect-site concentration (remifentanil 20 mcg/mL; ng/mL) | | 3.20 [2.02;4.01] | | 3.21 [2.03;4.01] | | 2.51 [1.55;4.00] | | 0.063 | | 4,644 | |
| Plasma concentration (propofol 20 mg/mL; mcg/mL) | | 3.00 [2.70;3.50] | | 3.00 [2.70;3.50] | | 2.69 [2.00;3.00] | | <0.001 | | 3,405 | |
| Infused volume (propofol 20 mg/mL; mL) | | 36.6 [25.6;51.9] | | 36.6 [25.6;52.0] | | 33.0 [19.1;49.4] | | 0.271 | | 3,409 | |
| Infused volume (remifentanil 20 mcg/mL; mL) | | 30.9 [20.4;49.3] | | 30.9 [20.4;49.3] | | 31.8 [17.6;44.1] | | 0.402 | | 4,642 | |
| Target concentration (remifentanil 20 mcg/mL; ng/mL) | | 3.20 [2.00;4.00] | | 3.20 [2.00;4.00] | | 2.50 [1.40;4.00] | | 0.081 | | 4,647 | |
| Plasma concentration (remifentanil 20 mcg/mL; ng/mL) | | 3.18 [2.01;4.01] | | 3.20 [2.01;4.01] | | 2.50 [1.20;4.00] | | 0.040 | | 4,642 | |
| Infusion rate (remifentanil 20 mcg/mL; mL/hr) | | 20.3 [12.7;27.0] | | 20.3 [12.7;27.0] | | 16.0 [7.63;24.7] | | 0.143 | | 4,649 | |
| Effect-site concentration (propofol 20 mg/mL; mcg/mL) | | 3.00 [2.70;3.50] | | 3.00 [2.70;3.50] | | 2.70 [2.00;3.00] | | <0.001 | | 3,408 | |
| Target concentration (propofol 20 mg/mL; mcg/mL) | | 3.00 [2.70;3.50] | | 3.00 [2.70;3.50] | | 2.60 [2.00;3.00] | | <0.001 | | 3,404 | |
| *BIS Vista EEG Monitor* |  | |  | |  | |  | |  | |  |
| Bispectral index value (-) | | 40.7 [36.7;44.7] | | 40.8 [36.8;44.7] | | 40.2 [34.0;42.3] | | 0.042 | | 5,571 | |
| Spectral edge frequency (Hz) | | 15.3 [13.9;16.5] | | 15.3 [13.9;16.5] | | 13.5 [11.6;15.2] | | <0.001 | | 5,487 | |
| EEG wave from channel 1 (uV) | | 19.4 [9.65;24.5] | | 19.5 [9.65;24.5] | | 19.1 [10.1;24.1] | | 0.973 | | 5,145 | |
| Suppression ratio (%) | | 0.0 [0.0;0.0] | | 0.0 [0.0;0.0] | | 0.0 [0.0;1.6] | | <0.001 | | 5,483 | |
| EEG wave from channel 2 (uV) | | 16.5 [8.80;22.0] | | 16.5 [8.80;22.0] | | 17.3 [8.56;22.2] | | 0.646 | | 4,467 | |
| Electromyography power (dB) | | 26.6 [25.9;27.4] | | 26.6 [25.9;27.4] | | 26.3 [25.6;27.8] | | 0.178 | | 5,491 | |
| Total power (dB) | | 62.1 [59.9;64.5] | | 62.1 [59.9;64.5] | | 60.8 [57.9;62.9] | | 0.004 | | 5,461 | |
| Signal quality index (%) | | 92.3 [86.5;96.2] | | 92.3 [86.5;96.2] | | 94.9 [84.6;97.4] | | 0.091 | | 5,571 | |

**Supplementary Table SM3.** Summary metrics of monitor variables. Unadjusted group comparisons with respect to the primary outcome in-hospital mortality are shown for exploratory purposes. Data availability is shown for each variable.

| **Learner** | **Domain** | **AUROC** | **AUPRC** | **Brier score** | **Scaled Brier Score** |
| --- | --- | --- | --- | --- | --- |
| Logistic Regression | All Data | 0.61 (0.50 - 0.76) | 0.04 (0.01 - 0.18) | 0.02 (0.01 - 0.04) | -1.21 (-3.40 - -0.39) |
| Random Forest |  | 0.73 (0.63 - 0.83) | 0.21 (0.07 - 0.38) | 0.01 (0.01 - 0.01) | 0.08 (-0.05 - 0.16) |
| Gradient Boosting Machine |  | 0.75 (0.64 - 0.85) | 0.18 (0.05 - 0.35) | 0.01 (0.01 - 0.01) | 0.06 (-0.20 - 0.22) |
| Neural Network |  | 0.68 (0.55 - 0.79) | 0.07 (0.02 - 0.20) | 0.01 (0.01 - 0.02) | -0.30 (-0.98 - -0.02) |
| Stacked Ensemble |  | 0.74 (0.62 - 0.84) | 0.18 (0.05 - 0.33) | 0.01 (0.01 - 0.01) | 0.08 (-0.15 - 0.20) |
| Logistic Regression | Preoperative   (Demographics, Comorbidities & Intervention) | 0.74 (0.63 - 0.85) | 0.19 (0.06 - 0.34) | 0.01 (0.01 - 0.01) | 0.08 (-0.08 - 0.20) |
| Random Forest |  | 0.77 (0.65 - 0.86) | 0.20 (0.07 - 0.34) | 0.01 (0.01 - 0.01) | 0.09 (-0.03 - 0.14) |
| Gradient Boosting Machine |  | 0.75 (0.63 - 0.85) | 0.18 (0.05 - 0.32) | 0.01 (0.01 - 0.01) | 0.08 (-0.08 - 0.19) |
| Neural Network |  | 0.70 (0.56 - 0.81) | 0.13 (0.03 - 0.29) | 0.01 (0.01 - 0.02) | 0.01 (-0.87 - 0.13) |
| Stacked Ensemble |  | 0.73 (0.62 - 0.84) | 0.13 (0.04 - 0.29) | 0.01 (0.01 - 0.01) | 0.04 (-0.19 - 0.16) |
| Logistic Regression | Preoperative   (Laboratory) | 0.78 (0.69 - 0.86) | 0.10 (0.04 - 0.18) | 0.01 (0.01 - 0.01) | 0.01 (-0.13 - 0.08) |
| Random Forest |  | 0.74 (0.63 - 0.84) | 0.25 (0.09 - 0.39) | 0.01 (0.01 - 0.01) | 0.12 (-0.04 - 0.24) |
| Gradient Boosting Machine |  | 0.74 (0.64 - 0.83) | 0.17 (0.06 - 0.33) | 0.01 (0.01 - 0.01) | 0.07 (-0.11 - 0.20) |
| Neural Network |  | 0.77 (0.62 - 0.86) | 0.09 (0.04 - 0.22) | 0.01 (0.01 - 0.01) | 0.04 (-0.07 - 0.11) |
| Stacked Ensemble |  | 0.75 (0.65 - 0.84) | 0.19 (0.06 - 0.34) | 0.01 (0.01 - 0.01) | 0.10 (-0.08 - 0.21) |
| Logistic Regression | Intraoperative   (Monitors) | 0.67 (0.55 - 0.77) | 0.06 (0.02 - 0.18) | 0.01 (0.01 - 0.01) | -0.20 (-0.51 - 0.02) |
| Random Forest |  | 0.68 (0.57 - 0.79) | 0.12 (0.03 - 0.25) | 0.01 (0.01 - 0.01) | 0.03 (-0.12 - 0.11) |
| Gradient Boosting Machine |  | 0.69 (0.58 - 0.80) | 0.09 (0.02 - 0.23) | 0.01 (0.01 - 0.01) | -0.02 (-0.22 - 0.12) |
| Neural Network |  | 0.63 (0.51 - 0.75) | 0.03 (0.01 - 0.12) | 0.01 (0.01 - 0.02) | -0.32 (-0.96 - -0.03) |
| Stacked Ensemble |  | 0.68 (0.57 - 0.78) | 0.09 (0.02 - 0.21) | 0.01 (0.01 - 0.01) | 0.02 (-0.15 - 0.11) |
| Logistic Regression | Intraoperative   (Laboratory, Drugs & Fluids) | 0.55 (0.41 - 0.67) | 0.06 (0.01 - 0.15) | 0.01 (0.01 - 0.01) | -0.05 (-0.24 - 0.07) |
| Random Forest |  | 0.62 (0.51 - 0.72) | 0.09 (0.02 - 0.24) | 0.01 (0.01 - 0.01) | 0.02 (-0.15 - 0.08) |
| Gradient Boosting Machine |  | 0.61 (0.48 - 0.70) | 0.05 (0.01 - 0.16) | 0.01 (0.01 - 0.01) | -0.01 (-0.13 - 0.08) |
| Neural Network |  | 0.53 (0.41 - 0.63) | 0.02 (0.01 - 0.12) | 0.01 (0.01 - 0.02) | -0.27 (-1.19 - 0.01) |
| Stacked Ensemble |  | 0.59 (0.45 - 0.71) | 0.07 (0.01 - 0.19) | 0.01 (0.01 - 0.01) | 0.02 (-0.19 - 0.09) |

**Supplementary Table SM4.** Performance metrics for each learner and predictors from different domains. Mean and 95%-confidence intervals are shown.
